# Comparison of Fibrin Glue With Conventional Suturing in Peripheral Nerve Repairs: A Study of Sensory and Motor Outcomes

**DOI:** 10.7759/cureus.63111

**Published:** 2024-06-25

**Authors:** Ansarul Haq, Veena Kumari, Varun H Kashyap, Jatin Goel

**Affiliations:** 1 Burns and Plastic Surgery, All India Institute of Medical Sciences, Patna, IND

**Keywords:** fibrin glue, nerve repair, brachial plexus, cooptation, median nerve, ulnar nerve, sutures, microsutures, magnifying loupe, microsurgery

## Abstract

Background: Nerve injuries have traditionally been repaired with sutures, and this method is considered the gold standard technique in the management of nerve injuries. However, fibrin glue has recently become a promising tool for repairing nerve injuries and has advantages including ease of usability, atraumatic application technique, and decreased co-optation time of the nerves. This study aims to clinically evaluate the efficacy of nerve repair with fibrin glue compared with the usual suture technique in terms of sensory and motor outcomes.

Methods: A total of 80 patients were included in the study; 50 patients underwent primary nerve repair, and 30 patients underwent Oberlin’s repair. These subsets were randomly divided into two groups in which the nerves were repaired with microsutures in one group and fibrin glue in the other group.

Results: In the comparison of fibrin glue with microsutures, there were no significant differences between the two groups in the 2-point discrimination (2PD) test, Semmes-Weinstein test, motor function, and Disabilities of the Arm, Shoulder, and Hand (DASH) questionnaire scores. However, the co-optation times were significantly shorter with fibrin glue than with microsutures.

Conclusion: Based on our findings, nerve repair with fibrin glue is as effective as microsutures in terms of sensory and motor recovery and has added advantages of ease of usability and shorter repair times. Therefore, fibrin glue may be an effective alternative to sutures in nerve repair.

## Introduction

For effective repair of peripheral nerves, an accurate adaptation of the fascicles of the severed nerves is necessary. Inaccurate co-optation can prevent the passage of regenerating axons to the distal perineural tubes. Stabilization of the peripheral nerve ends with fibrin was first described by Young and Medawar [1] in 1940 and Tarlow [2] in 1943. Despite its early description, the use of fibrin as a nerve sealant was uncommon until the first commercially available fibrin glue, Tisseel (Baxter, Deerfield, IL, USA), was introduced in the 1970s [3]. Before its introduction, the only effective method of nerve repair was by microsurgical suturing [4,5]; however, evidence suggests that this technique may cause some damage to neurons and inflammatory reactions that may hinder nerve regeneration [6-9].

Fibrin glue contains fibrinogen, plasma proteins, and factor XIII mixed with thrombin, calcium chloride, and aprotinin. This mixture simulates the end stages of the clotting cascade and forms a substance resembling a physiological blood clot [10]. This clot acts as an adhesive sealant that holds the cut ends of the nerves together [3,7]. The structural integrity of the co-optation is preserved for three weeks by the antifibrinolytic properties of the compound; during this period, the adhesive sealant protects the nerve repair from scar tissue formation and allows for healing to occur within the epineurium [10,11]. The major advantages of fibrin glue are a reduced anastomotic time, minimal perioperative trauma to nerve endings, and decreased fibrosis and inflammation during healing of the repair, resulting in decreased scar tissue formation and eventually a better repair [12-14]. A sutureless repair has the additional advantage of overcoming the problem of rupture of the repair that is associated with sutures [7].

Although Tisseel was introduced more than 40 years ago, there are very few reports of its use in the repair of peripheral nerves. Among previous studies, the majority report its use in rats and compare it with the microsurgical suturing technique [6,8,14]. While a few studies have reported the use of fibrin glue for nerve repairs in humans, only one study has compared the efficacy of fibrin glue versus the microsuture technique in human peripheral nerve repairs [15]. The purpose of this study is to analyze and compare the outcomes between the conventional suture technique and the fibrin glue technique in terms of both motor and sensory nerve recovery.

## Materials and methods

This study is a double-blinded randomized controlled trial conducted in adherence to the Consolidated Standards of Reporting Trials (CONSORT) guidelines, approved by the All India Institute of Medical Sciences (AIIMS), Patna, India (approval no. RD/AIIMS/Pat//RAC-125), and performed per the principles of the Declaration of Helsinki. Written informed consent for surgery was duly provided by all patients included in this study. In calculating the sample size, with beta = 0.95 and alpha = 0.05, at least 80 participants were required (PASS version 15.0; NCSS LLC, Kaysville, UT, USA). A total of 80 patients were included in this study at our tertiary referral center during the period between January 2018 and January 2022. Patients with peripheral nerve injuries as well as brachial plexus injuries were included in the study. Patients with peripheral nerve injuries underwent surgery within two weeks, and those with brachial plexus injuries were evaluated and underwent surgeries within six months. Patients with brachial plexus injuries were evaluated with MRI and nerve conduction studies as well as clinical examinations, and only those patients in whom Oberlin’s I and II transfer procedures were possible, were included.

Inclusion and exclusion criteria

All adult patients aged between 18 and 60 years with peripheral nerve injuries of the upper limb presenting within two weeks and patients with brachial plexus injuries for which Oberlin’s I and II transfer had to be performed, were included in our study. Patients presenting with delayed peripheral nerve injuries and those requiring nerve grafts were excluded from the study. Patients with pan-brachial plexus injuries and those for whom Oberlin’s transfer was not possible, were also excluded.

Surgical procedure

**Figure 1:**
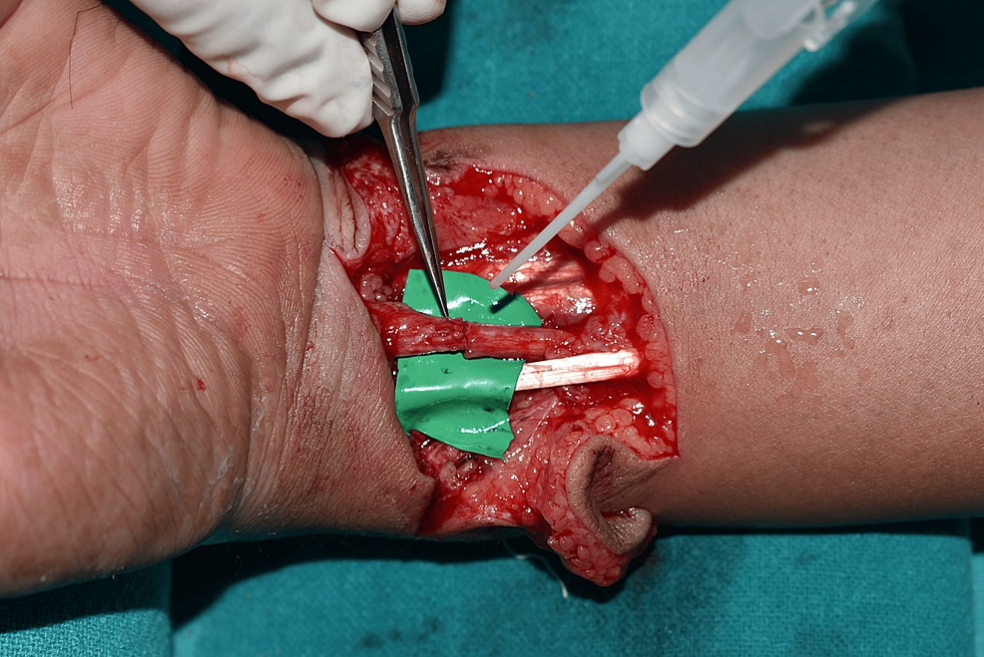
Intraoperative image demonstrating the repair of the median nerve with fibrin glue

The patients underwent surgery performed under tourniquet control. After exposure of the injured nerve ends, external neurolysis was performed. If a neuroma or scar was present, it was excised, and the ends were trimmed up until healthy fascicles were visualized. The fascicles from both ends were properly oriented and aligned, and an epineural repair was performed with 10-0 nylon suture or fibrin glue application as shown in Figure 1.

**Figure 2:**
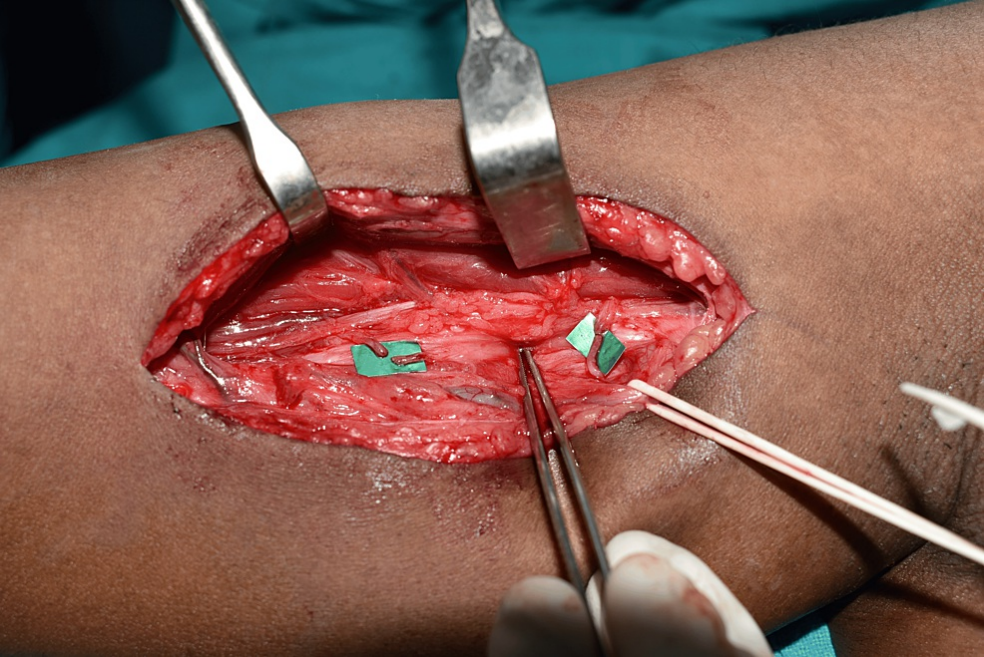
Intraoperative image showing the application of fibrin glue in the co-optation of the ulnar fascicle to the branch of the biceps and the median fascicle to the branch of the brachialis

In patients with brachial plexus injuries with an incision on the medial arm, the musculocutaneous, median, and ulnar nerves were exposed. Oberlin’s transfer was performed with the co-optation of a branch of the median nerve with a branch to the brachialis muscle and a branch from the ulnar nerve to a branch of the biceps muscle. The repair was performed with either 10-0 nylon sutures or fibrin glue as shown in Figure 2. If required, a spinal accessory to suprascapular nerve transfer was also performed along with Oberlin’s transfer either by the anterior or posterior approach.

All dissections and fibrin glue applications were performed under loupe magnification, and all nerve suturing was performed under a microscope. In the fibrin glue groups, co-optation of nerve fascicles was done only with fibrin glue without employing any sutures. Similar brands of suture material (Ethicon; Johnson & Johnson MedTech, New Brunswick, NJ, USA) and fibrin glue (Tisseel; Baxter) were used in all cases. If there were associated tendon or vessel injuries, they were repaired in the standard manner. After closure, the limb was dressed and splinted in the desired position for four weeks.

Patients were randomized into two groups by block randomization, and as a result, a total of four groups were formed. Group 1a included peripheral nerve injuries treated with approximation by primary suturing with 10-0 nylon suture. Group 1b had peripheral nerve injuries treated with approximation with fibrin glue. Group 2a featured Brachial plexus injuries treated with Oberlin’s transfer I and II repaired with a 10-0 nylon suture. Group 2b involved brachial plexus injuries treated with Oberlin’s transfer I and II repaired with fibrin glue.

This study is a double-blinded study with blinding of the patients as well as the observers. The blinding of surgeons was not possible in our study. Information regarding the patient's age, etiology of the injury, duration of the injury, nerve(s) involved, repair undertaken, presence of nerve defects, number of nerve cables applied, the distance between the proximal nerve end and the tip of the middle finger, presence of an associated vascular injury, and comorbidities were recorded. The patients included in the study were matched with a randomly selected cohort of control patients with nerve injuries according to the patient's age, duration of the injury, type of nerve injury, and presence of a nerve gap.

Patients were followed up for two years or until complete recovery at intervals of three months. Eighteen months after surgery, the 2-point discrimination (2PD), Semmes-Weinstein test, and the Medical Research Council (MRC) scale for muscle strength were used for the final evaluation of sensory and motor outcomes for peripheral nerve injuries (Tables 1-2). Only motor recovery was evaluated in patients in whom Oberlin’s transfer was performed.

**Table 1:** Scales used to estimate sensory recovery 2PD: 2-point discrimination

Sensory recovery scales	Values
The 2PD test	2PD distance
Normal	0–5 mm
Fair	6–10 mm
Poor	11–15 mm
Very poor	> 15 mm
Semmes-Weinstein monofilament test	Monofilament size (target force)
0	Not testable
1	Filament 6.65
2	Filament 4.56 (4 g)
3	Filament 4.31 (2.0 g)
4	Filament 3.61 (0.2 g)
5	Filament 2.83 (0.05 g)

**Table 2:** Scale to estimate motor recovery MRC: Medical Research Council

Power grading of the affected muscles (MRC scale)	Degree of strength
Grade 0	Complete paralysis
Grade 1	Flicker of contraction present
Grade 2	Active movement with gravity eliminated
Grade 3	Active movement against gravity
Grade 4	Active movement against gravity and some resistance
Grade 5	Normal power

The 2PD test was done in a standard manner at the fingertips. The MRC grading was performed by assessing the muscle strength of the first dorsal interossei to evaluate the ulnar nerve and the flexor pollicis brevis to evaluate the median nerve. An average of three readings was taken to evaluate the sensory tests. The Disabilities of the Arm, Shoulder, and Hand (DASH) questionnaire, which consists of 30 questions, was used to subjectively quantify the patient’s disability [16,17]. This tool has been previously used to evaluate peripheral nerve injuries as well as brachial plexus injuries [18-20].

Statistical analysis

Statistical analysis was performed using SPSS Statistics version 25.0 (IBM Corp., Armonk, NY, USA). The Kolmogorov-Smirnov and Shapiro-Wilk tests denoted that the scores of the 2PD test, Semmes-Weinstein test, and DASH questionnaire as well as the operative times deviated from normality. Therefore, the Mann-Whitney test was used for intergroup comparisons. The power of the study was taken to be 80%, and all statistical significances were set at p < 0.05.

## Results

Demographic characteristics

The demographic characteristics of all 80 patients with a mean follow-up of 18 months are presented in Table 3. The overall mean patient age was 31.64±11.02 years. Of 80 patients, 62 (77.5%) were male. The mean duration of the injury was 6.6±2.2 days in the peripheral nerve injury group and 11.2±10.2 days in the Oberlin group. The main mechanism of peripheral nerve injury was injury with a sharp object in 86.67% of cases, while traction injury following road traffic accident (RTA) was the cause in all (100%) patients with brachial plexus injuries.

**Table 3:** Demographic data of the study participants in each group The groups were compared using the Mann-Whitney test, and p < 0.05 was considered significant. RTA: Road traffic accident, DM: Diabetes mellitus, HTN: Hypertension

Groups	Group 1a (n = 25)	Group 1b (n = 25)	Group 2a (n = 15)	Group 2b (n = 15)
Age in years (mean±SD)	30.80±12.23	31.00±11.62	32.67±8.64	32.27±0.59
	-	p > 0.05	-	p > 0.05
Sex (M/F %)	72/28	80/20	86.7/13.3	73.3/26.7
Median n (%)	56	64	-	-
Ulnar n (%)	32	28	-	-
Median n + Ulnar n (%)	12	8	-	-
Musculocutaneous n	-	-	100	100
Sharp cut (%)	80	88	0	0
Crush/avulsion(%)	20	12	0	0
Traction injury following RTA (%)	0	0	100	100
Distance from the tip of the middle finger in cm (mean±SD)	22.8±6.8	24.4±8.4	-	-
	-	p > 0.05	-	-
Duration of injury in days (mean±SD)	6.8±2.1	6.4±2.4	108±11.6	116±8.9
	-	p > 0.05	-	p > 0.05
Associated vascular injury n (%)	3 (12)	2 (8)	0	0
Associated tendon injury n (%)	8 (32)	5 (20)	0	0
Comorbidities n (%) (DM, HTN)	3 (12)	2( 8)	3 (20)	1 (6.67)

Sensory evaluation

To measure tactile gnosis for patients who underwent peripheral nerve repair, a static 2PD test was performed. A 2PD test score < 5 was present in eight patients (32%) who underwent primary repair with sutures (group 1a), while it was present in seven patients (28%) who underwent primary repair with fibrin glue (group 1b). A 2PD test score of more than 15 mm was present in one patient in each group. Statistical analysis of both of these groups revealed a p-value that was greater than 0.05; therefore, the null hypothesis was retained (Table 4). Approximately 72% of patients in group 1a and 64% of patients in group 1b had a score of 4 on the Semmes-Weinstein monofilament test. Pain with disturbed function (a score of 1) was observed in four (16%) patients in group 1a, and five (20%) patients in group 1b (Table 4). Statistical analysis revealed no differences in the intergroup comparison (p > 0.05).

**Table 4:** Evaluation of sensory and motor outcomes, co-optation times, and DASH scores. Fisher’s exact test was used to compare sensory and motor outcomes, and the Mann-Whitney U test was used to compare the co-optation times and DASH scores. A p-value < 0.05 was considered significant. 2PD: 2-point discrimination; DASH: Disabilities of the Arm, Shoulder, and Hand; MRC: Medical Research Council

2PD test (mm)	Group 1a (n=25)	Group 1b (n=25)		
< 5	8 (32%)	7 (28%)	-	-
5–10	14(52%)	14(56%)	-	-
10–15	2 (8%)	3(12%)	-	-
>15	1 (4%)	1(4%)	-	-
-	-	p > 0.05	-	-
Semmes-Weinstein test score	Group 1a (n = 25)	Group 1b (n = 25)	-	
1	0	0	-	-
2	2 (8%)	3 (12%)	-	-
3	5 (20%)	6 (24%)	-	-
4	13 (52%)	10 (40%)	-	-
5	5 (20%)	6 (24%)	-	-
-	-	p > 0.05	-	-
MRC score	Group 1a (n = 25)	Group 1b (n = 25)	Group 2a (n = 15)	Group 2b (n = 15)
1	0	1 (4%)	0	0
2	4 (16%)	3 (12%)	2 (13.3%)	2 (13.3%)
3	4 (16%)	4 (16%)	5 (33.3%)	4 (26.7%)
4	13 (52%)	12 (48%)	8 (53.3%)	9 (60%)
5	4 (16%)	5 (20%)	0	0
-	-	p > 0.05	-	p > 0.05
Co-optation time (min)	Group 1a (n=25)	Group 1b (n=25)	Group 2a (n=15)	Group 2b (n=15)
Mean±SD	21.04±8.06	9.92±6.57	14.93±4.77	10.40±3.40
Min–max	8–40	4–28	10–24	6–20
-	-	p < 0.05	-	p < 0.05
DASH score	Group 1a (n=25)	Group 1b (n=25)	Group 2a (n=15)	Group 2b (n=15)
Mean±SD	25.04±15.65	22.88±14.89	38.13±12.15	35.87±15.48
Min–max	6–88	8–74	10–90	8–72
-	-	p > 0.05	-	p > 0.05

Motor function evaluation

In terms of motor recovery, 68% of patients had a satisfactory power of 4 in groups 1a and 1b. In patients with brachial plexus injuries, 53.3% of patients who underwent suture repair and 60% of those who underwent fibrin glue repair had a power of 4. Statistical analysis revealed that all the groups were comparable (p > 0.05) (Table 4).

Co-optation time

The mean co-optation time for primary nerve repairs was 21.04±8.06 minutes for repair by sutures and 9.92±6.57 minutes for repair by fibrin glue. For brachial plexus repairs, the mean co-optation time was 14.93±4.77 minutes for suture repair and 10.40±3.40 minutes for repair with fibrin glue (Table 4). In the statistical analysis, the p-value was < 0.05, and the co-optation times were significantly shorter in the groups in which fibrin glue was used for nerve repairs (groups 1b and 2b).

DASH scores

On comparison of the DASH scores, the mean DASH score was 25.04±15.65 for group 1a compared with 22.88±14.89 for group 1b. The mean DASH score for group 2a was 38.13±12.15, while it was 35.87±15.48 for group 2b. For the intergroup comparisons, the p-value was > 0.05. Higher DASH scores were found in patients with brachial plexus injuries who had concomitant upper trunk shoulder involvement (Table 4).

Confounding patient factors

Age

**Figure 3:**
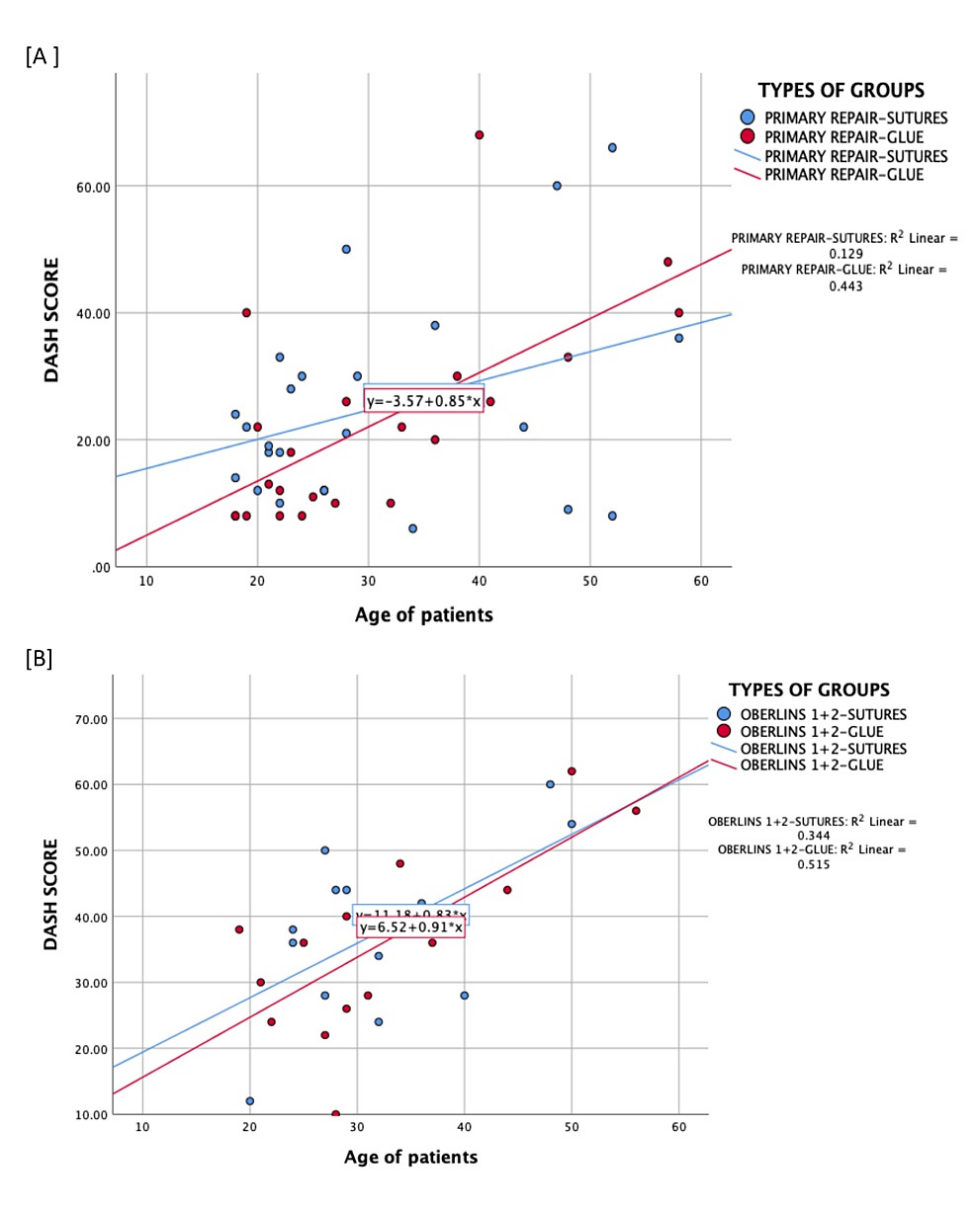
Scatterplot showing the correlation between age and DASH scores in the primary repair groups A: Graph depicting groups 1a and 1b; B: Graph depicting Oberlin groups, namely groups 2a and 2b DASH: Disabilities of the Arm, Shoulder, and Hand

A positive correlation was found between the patient's age and DASH scores, as depicted in Figure 3. The association was significant with Pearson’s correlation of 0.506 and p < 0.05. Although the DASH scores were higher in older patients, the trend was similar in the comparative groups.

Comorbidities

**Figure 4:**
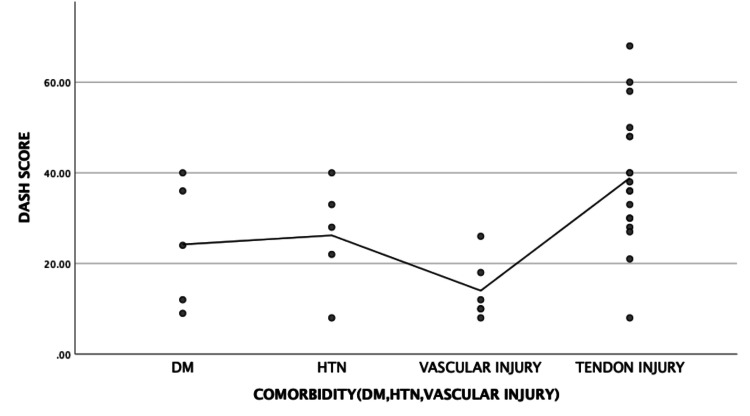
Graph showing the correlation between comorbidities and DASH scores DASH: Disabilities of the Arm, Shoulder, and Hand, DM: Diabetes mellitus, HTN: Hypertension

A similar positive correlation was found between the concomitant presence of tendon injury and increased DASH scores. However, the DASH scores were not significantly increased with comorbidities of diabetes mellitus, hypertension, or vascular injuries (Figure 4).

## Discussion

Fibrin glue was first introduced in the 1970s, and since then, its use has been increasing [3]. Fibrin glue is a compound that contains plasma proteins and their plasma activators in high concentrations. The commercially available fibrin glue, Tisseel (Baxter), which we used in this study, contains two kits. The first kit contains lyophilized protein concentrate (fibrinogen, plasma fibronectin), plasminogen, factor XIII, and aprotinin solution. The second kit contains lyophilized thrombin and calcium chloride. The lyophilized protein concentrate is reconstituted in aprotinin solution in the first kit, while the lyophilized thrombin is reconstituted in calcium chloride solution in the second kit. These two kits are mounted over a syringe with a common outlet, which allows the mixing of both solutions just before application. Upon expulsion from the syringe, these mixtures form a viscous solution that quickly sets to form a whitish plug that seals the nerve ends. The clot formed by Tisseel or fibrin glue is similar to a physiological clot [21,22].

Before the advent of fibrin glue, suture repair was the gold standard in the repair of peripheral nerves. Various studies have demonstrated the efficacy of fibrin glue in animal models. Repairing with fibrin glue has shown many advantages in nerve regeneration, such as reduced inflammation and fibrosis, resulting in a shortened recovery time due to decreased neural scar tissue [3-6,8,14]. Suture repair has been thought to be a hindrance to the growing axons and causes ischemia to the growing nerves by compressing the fascicles [23]. While these advantages of fibrin glue have been demonstrated in the literature, these previous studies were all limited to animal models. A preliminary clinical report published in 1983 evaluating 56 peripheral nerve repairs using human fibrin is perhaps the only study to compare fibrin glue to sutures in human nerve repair [15].

In our study, many variables were evaluated to compare fibrin glue with the conventional suturing method in primary nerve repair as well as in Oberlin’s transfer. Statistical tests were not applied in cases in which nerve grafts were used due to the small number of cases. Overall, the study population was young, with a mean age of 31.64±11.02 years with a random distribution across all of the study groups. Sensory evaluation of the study groups was performed with the 2PD test and Semmes-Weinstein test. There were no statistically significant differences between the groups in any of these tests. Motor function was evaluated with the MRC grading chart, which again showed no differences between the groups. Based upon the statistical analyses, the null hypothesis was retained in all tests, and there were no differences in terms of sensory and motor recovery of peripheral nerves repaired either by suture or fibrin glue. There were some confounding factors in our study, such as the association of age and concomitant tendon injury with increased DASH scores, but both of these variables were equally distributed across the groups.

An important observation in our study was the significant difference between the co-optation time of the nerves between suture repair and fibrin glue repair across all subgroups. The greatest advantage of fibrin glue over sutures is the reduced time required for co-optation. Although the packaging must be thawed for approximately 30 minutes before application, it takes only a few minutes to prepare the glue mixture and a few seconds for the glue to set over the opposed nerve ends. Fibrin glue can be easily applied with surgical loupes and does not require an operating microscope. On the other hand, suture repair requires very fine 10-0/11-0 sutures, delicate handling, expertise in microsurgery, and the use of a microscope. Various previous studies have demonstrated that fibrin glue repair is easier as well as quicker [8,9,14,24,25]. In a study by Ornelas et al., the average time required for nerve repair with human fibrin was 57 seconds, whereas for rat sciatic nerve repair with sutures was 180 seconds.

There has been some criticism regarding the initial tensile strength offered by fibrin glue repair; some studies have cited difficulty in initially holding the nerve ends together [4,26] and suggested that fibrin glue repair is more prone to disruption in cases of sudden movements. However, two studies opposed this opinion. Issacs et al. concluded that fibrin had greater resistance to disruption than sutures and no difference in the peak loads [3]. In the study by Nishimura et al., they concluded that although fibrin glue repair was weak in the first seven days, its strength was equal to that of suture repair afterward; ultimately, in the long term, fibrin glue repair had final resistance that was greater than that of conventional suture repair [24].

Limitations of the study

The main limitation of our study was the small number of cases. However, other biases were reduced as all the patients underwent surgery performed by the same surgeons. Furthermore, the fibrin glue and sutures used were of similar brands in all the patients.

## Conclusions

Based on our findings, fibrin glue is almost as effective for peripheral nerve repair as the conventional suture technique in terms of sensory and motor recovery as well as postoperative functional recovery. Added advantages of fibrin glue are the ease of application, decreased requirement for microsurgical skills, significantly decreased operative time, and possibly better outcomes due to reduced inflammation during nerve healing. However, as mentioned in previous studies, precautions should be taken to prevent initial disruption.
